# Vitamin D protects podocytes from autoantibodies induced injury in lupus nephritis by reducing aberrant autophagy

**DOI:** 10.1186/s13075-018-1803-9

**Published:** 2019-01-11

**Authors:** Qi Yu, Yingjin Qiao, Dongwei Liu, Fengxun Liu, Congcong Gao, Jiayu Duan, Lulu Liang, Xueqi Di, Yi Yuan, Yukui Gao, Siwan Cui, Yilu Qin, Tianfang Li, Zhaohui Zheng, Zhangsuo Liu

**Affiliations:** 1 Department of Nephrology, Research Institute of Nephrology, Key Laboratory of Precision Diagnosis and Treatment for Chronic Kidney Disease in Henan Province, Core Unit of National Clinical Medical Research Center of Kidney Disease, The First Affiliated Hospital of Zhengzhou University, Nephrology, 1 Easten Jianshe Road, Zhengzhou, 450052 Henan People’s Republic of China; 2 Department of Rheumatology, The First Affiliated Hospital of Zhengzhou University, 1 Easten Jianshe Road, Zhengzhou, 450052 Henan People’s Republic of China; 3 Institute of Nephrology, Blood Purification Center, The First Affiliated Hospital of Zhengzhou University, 1 Easten Jianshe Road, Zhengzhou, 450052 Henan People’s Republic of China; 4 Department of Urology, The First Affiliated Hospital of Zhengzhou University, 1 Easten Jianshe Road, Zhengzhou, 450052 Henan People’s Republic of China

**Keywords:** Vitamin D, Podocyte, Lupus nephritis, Autophagy

## Abstract

**Subject:**

The aim of this study was to investigate whether vitamin D plays a protective role in podocyte injury induced by autoantibodies purified from the serum of patients with lupus nephritis (LN) via reducing aberrant autophagy.

**Methods:**

Autophagic activities of renal tissues of patients with LN were evaluated under transmission electronic microscope (TEM). Immunoglobulin G (IgG) from patients with LN was purified to induce human podocyte injury, and the role of vitamin D in injury was observed. Podocytes were observed under TEM, autophagic activity was evaluated by western blot analysis and quantitative real-time polymerase chain reaction, and mRFP-GFP-LC3B adenovirus was infected into human podocytes *in vitro*.

**Results:**

Significantly higher autophagic levels were observed in patients with LN (*P* <0.05), and apparently greater autophagic levels in podocytes were shown (*P* <0.05). Among different classifications of LN, class V (n = 5), III + V (n = 5), and IV + V (n = 5) gained higher autophagic levels than class III (n = 5) and IV (n = 5). Induced autophagy, which was evident by increased LC3B-II and Beclin 1 level, caused consumption of p62, more autophagosomes observed under TEM, and more LC3B dots observed under confocal microscope in the IgG group, along with decreased nephrin expression, which suggests podocyte injury. Reduction of autophagy as well as alleviated podocyte injury was observed in the IgG+ vitamin D group.

**Conclusion:**

This study demonstrates that vitamin D plays a protective role in podocyte injury induced by autoantibodies from patients with LN and appears to be a novel therapy target in LN.

## Highlights

- This study demonstrates that autophagy is activated in the kidney tissue of patients with lupus nephritis, especially in podocytes.
- Observation at the autophagic level in patients with lupus nephritis proved variation and correlation with 25(OH)D3 concentration.
- In *in vitro* assays, IgG purified from patients with lupus nephritis could induce autophagy in human podocytes, which causes podocyte injury. The addition of vitamin D could reduce aberrant autophagy, which alleviated podocyte damage.
- In *in vitro* experiments, autophagy activation was positively associated with podocyte injury, which was induced by IgG (1.5 mg/mL) purified from sera of patients with lupus nephritis.

## Introduction

Systemic lupus erythematosus (SLE) is an autoimmune disease with potential involvement of virtually any organ of the body [1]. Lupus nephritis (LN) remains one of the most common and severe complications. Despite intensive studies, the pathogenesis of LN is not completely understood, and the development of novel therapeutics with an excellent safety profile and efficacy is still an unmet medical need. Podocytes are highly specialized epithelial cells and their slit diaphragms are the important part of the filtration barrier of glomeruli. The effacement of the foot processes caused by podocyte injury has been associated with the development of proteinuria [2]. Preventing podocyte injury may help identify new treatment targets to improve the renal prognosis in patients with LN [3].

In addition to its critical role in maintaining calcium and phosphorus homeostasis, recent data suggest that vitamin D insufficiency may play a role in the progression of SLE and nephropathy such as chronic kidney disease [4]. We previously showed that severe vitamin D deficiency increases the risk for moderate to severe disease activity [5]. Consistent with this, Petri et al. demonstrate that a 20-ng/mL increase in the 25(OH)D level is associated with a 21% decrease in the odds of having a high disease activity score and a 15% decrease in the odds of having clinically important proteinuria [6]. Moreover, vitamin D has been demonstrated to have a protective effect on podocyte injury via diverse mechanisms in proteinuric glomerular disease [7–11]. Collectively, these findings indicate that vitamin D deficiency is associated with nephropathy and the supplement of vitamin D may prevent renal involvement by alleviating podocyte injury.

Autophagy, an endogenous process necessary for the turnover of organelles, maintains cellular homeostasis and determines cell fate [12]. Under normal circumstances, the intracellular process of autophagy is tightly controlled. However, the autophagy process is dysregulated in autoimmune diseases, including SLE [12, 13]. Previous studies show that autophagy participates in the pathogenesis of LN [14] by damaging podocytes [15]. Previous observations suggest that vitamin D and its analog EB1098 may have a role in the treatment efficacy via regulation of autophagy [16, 17]. However, there is a paucity of data to demonstrate the mechanisms through which the autophagy functions in the protective role that vitamin D plays on podocytes in the pathogenesis of LN. The aim of our study was to investigate whether vitamin D can alleviate podocyte injury of patients with LN by regulating autophagy levels and play a protective role in podocytes.

## Material and methods

### Patient selection and renal biopsy

In all patients, LN was diagnosed on the basis of renal biopsies carried out at the Department of Nephrology, the First Affiliated Hospital of Zhengzhou University, Henan, China. The patients were selected by using the International Society of Nephrology/Renal Pathology Society (ISN/RPS) 2003 classification of LN [18]. Exclusion criteria include (1) coexistence of other autoimmune diseases such as rheumatoid arthritis, systemic sclerosis, and inflammatory myopathy; (2) coexistence of symptoms of obesity, diabetes mellitus, hepatitis B virus infection, hepatitis, malignancies, and patients undergoing continuous renal replacement therapy; and (3) pregnancy or lactation.

Altogether, 25 patients with LN (class III, n = 5; class IV, n = 5; class V, n = 5; class III + V, n = 5; class IV + V, n = 5) and 7 healthy volunteers were enrolled in this study. Healthy volunteers (with renal carcinoma) had no clinical features of kidney dysfunction and their glomeruli were pathologically normal. The ages of the patients ranged from 9 to 50 years (average age of 25.3). All patients were female. The study protocols were approved by the Human Subjects Committee of the First Affiliated Hospital of Zhengzhou University. All patients provided written informed consent.

Demographic, laboratory, and renal pathological data were recorded (Table 1). Demographic information included name, age, and gender. Laboratory data included blood urea nitrogen (BUN), serum creatinine (Scr), uric acid (UA), albumin, glomerular filtration rate (GFR), hemoglobin, urine erythrocyte and urine leukocyte, C-reactive protein (CRP), erythrocyte sedimentation rate (ESR), serum C3 and C4, and 24 h total protein in urine. Renal pathological indices included AIs (activity indices) and CIs (chronicity indices). Twenty serum samples from patients with LN were used in this study, and 30 serum samples from the healthy volunteers were used as controls.

**Table 1:** Demographic, clinical, and renal histopathology data of healthy controls (n = 7) and patients with lupus nephritis (n = 25)

Factors	Patients with lupus nephritis (n = 25)				
	III (n = 5)	IV (n = 5)	V (n = 5)	III + V (n = 5)	IV + V (n = 5)
Demographic data					
Gender female/male	5/0	5/0	5/0	5/0	5/0
Age	26.20 ± 15.42	28.20 ± 8.44	25.20 ± 13.59	22.80 ± 16.10	24.00 ± 12.98
Laboratory data					
BUN, mmol/L	6.80 ± 4.47	14.65 ± 10.81	4.36 ± 1.04	6.20 ± 2.07	7.78 ± 1.97
Scr, μmol/L	57.00 ± 22.85	151.80 ± 111.57	45.40 ± 7.96	57.08 ± 23.14	66.40 ± 6.58
UA, μmol/L	366.00 ± 68.75	466.60 ± 137.05	221.80 ± 45.45	298.40 ± 95.89	316.20 ± 87.76
Alb, g/L	29.84 ± 8.50	30.48 ± 7.10	21.04 ± 15.67	25.68 ± 8.29	25.78 ± 6.46
GFR, mL/min per 1.73 m^2^	122.75 ± 36.92	64.66 ± 48.66	135.16 ± 17.70	121.93 ± 39.07	111.15 ± 12.93
Hemoglobin, g/L	102.60 ± 14.10	96.40 ± 21.36	121.60 ± 16.52	121.93 ± 39.97	97.15 ± 12.93
24hTP, g	4.54 ± 3.90	3.31 ± 2.81	6.13 ± 4.91	2.99 ± 2.30	7.02 ± 3.39
RBC in urine, μL	20.80 ± 15.67	157.70 ± 244.21	11.20 ± 19.72	255.74 ± 457.50	63.32 ± 60.63
WBC in urine, μL	12.20 ± 5.22	195.90 ± 346.09	18.24 ± 29.73	19.76 ± 18.30	43.50 ± 37.45
CRP, mg/L	0.82 ± 1.03	2.71 ± 2.88	1.27 ± 1.10	26.44 ± 55.94	23.60 ± 3.68
ESR, mm/h	63.00 ± 42.03	37.20 ± 24.15	49.80 ± 34.27	42.60 ± 36.52	23.60 ± 9.07
Serum C3, g/L	0.58 ± 0.24	0.59 ± 0.386	1.00 ± 0.17	0.64 ± 0.22	0.67 ± 0.13
Serum C4, g/L	0.10 ± 0.07	0.10 ± 0.07	0.27 ± 0.09	0.17 ± 0.08	0.12 ± 0.08
Renal histopathology indices					
Al score	2 (2 4)	8 (8 15)	0 (0 0)	2 (0 5)	5 (3 6)
CI score	0 (0 4)	1 (0 3)	0 (0 0)	0 (0 1)	1 (1 2)
Autophagosome					
In podocyte	59.40 ± 15.50	33.00 ± 3.16	91.40 ± 43.18	86.00 ± 42.07	93.20 ± 39.90
In mesangial cell	35.60 ± 11.95	23.40 ± 11.15	22.20 ± 5.81	49.40 ± 34.69	53.80 ± 29.80
In endothelial cell	31.60 ± 4.67	17.20 ± 9.12	37.60 ± 16.10	53.60 ± 30.34	53.20 ± 18.019
In renal tubular epithelial cell	40.20 ± 21.46	30.20 ± 10.38	56.20 ± 32.57	43.20 ± 20.54	33.40 ± 14.57

Data are expressed as mean ± standard deviation or median, range, as appropriate. Abbreviations: *24hTP* 24 h total protein, *AI* active index, *Alb* serum albumin, *BUN* blood urea nitrogen, *CI* chronic index, *CRP* C-reactive protein, *ESR* erythrocyte sedimentation rate, *GFR* glomerular filtration rate, *RBC* red blood cells, *Scr* serum creatinine, *UA* urea acid, *WBC* white blood cells

Renal biopsy specimens were collected for measuring the level of autophagy by counting the number of autophagosomes under transmission electron microscopy (TEM). Serum samples were collected for immunoglobulin G (IgG) purification and then treated on cultured human podocytes.

### Measurement and definition of serum 25(OH)D3 levels

To avoid possible circadian variation, blood samples were collected after an overnight fasting between 8 and 9 a.m. Serum 25(OH)D3 levels of all patients were measured by using an electrochemiluminescence immuno-assay on an automated analyzer (ELECSYS-2010) with kits from Roche Diagnostics (Mannheim, Germany). This highly sensitive technique facilitates the detection of a very low concentration of 25(OH)D3 in sera. Serum 25(OH)D3 levels less than 30 and less than 15 ng/mL were defined as vitamin D insufficiency and vitamin D deficiency, respectively [19].

### IgG purification

ProteinIso Protein G Resin (TransGen Biotech, Beijing, China) was used for isolation and purification of IgG of patients with LN in accordance with the protocol of the manufacturer [20].

### Cell culture and treatment

Immortalized human podocytes (HPCs) were kindly provided by Rujun Gong of Brown University. These cells were cultured in RPMI 1640 medium (Gibco, part of Thermo Fisher Scientific, Waltham, MA, USA, USA) supplemented with 10% fetal bovine serum (FBS) (Gibco), 100 U/mL penicillin, and 100 μg/mL streptomycin (Geneview, Shanghai, China) at 37 °C with 5% CO_2_. Subsequent experiments were conducted when cells were fully differentiated. The cell treatments were as follows: (1) purified IgG from patients with LN alone (1.5 mg/mL), (2) 1,25-D3 (100 nM, Sigma-Aldrich) alone, and (3) purified IgG from patients with LN (1.5 mg/mL) plus 1,25-D3 (100 nM, Sigma-Aldrich) for 48 h.

### Transmission electron microscopy

TEM was performed with a JEOL JEM-1400 Plus transmission electron microscope (JEOL USA, Inc., Peabody, MA, USA). HPCs were trypsinized and the centrifuged cells were fixed with 2% glutaraldehyde for 12 h. After thorough wash with 0.1 M phosphate buffer, these cells were fixed with 1% OsO_4_ (in 0.1 M phosphate-buffered saline, or PBS) for 2 h. Then the samples were washed and stained with 3% aqueous uranyl acetate for 1 h. After washes, they were dehydrated with a graded acetone series and embedded in 812 resin (Canemco, 034).

The kidney specimens were fixed in glutaraldeyde immediately after biopsy by using protocols mentioned above. Ultrathin sections were cut with an ultramicrotome, stained with 2% (wt/vol) uranyl acetate and lead citrate, and examined with a JEOL JEM-1400 Plus transmission electron microscope. Autophagosomes in podocytes were identified according to the morphology described previously [21]. Under 5000× high-power field, each kidney tissue sample was examined and copper mesh was randomly selected to count the number of autophagosomes and autophagolysosomes in podocytes (including foot processes), mesangial cells, and endothelial cells of a glomeruli as well as in the renal tubular epithelial cells of the renal tubules. The area of a copper mesh is approximately 7065 square millimeters. After counting by more than two certified experienced pathologists, statistics analysis was performed.

### Western blotting

Cultured cells were lysed in radioimmunoprecipitation assay (RIPA) buffer supplemented with protease inhibitors. Samples were subjected to immunoblot analysis as previously described. The following antibodies were used: beclin 1 and p62 (Cell Signaling Technology, Danvers, MA, USA), nephrin and LC3B (Abcam, Shanghai, China), and GAPDH (Zhixian, Hangzhou, China). Secondary antibodies were obtained from Dingguo (Beijing, China).

### RNA extraction and real-time qRT-PCR

Total RNA was extracted by using an RNA Extraction Kit (Qiagen, Hilden, Germany) in accordance with the protocol of the manufacturer. Subsequently, the complementary DNA (cDNA) was synthesized by using a RevertAid First Strand cDNA Synthesis Kit (Thermo Fisher Scientific, Shanghai, China). In addition, the relative levels of the target gene mRNA transcript were measured by using quantitative reverse transcription-polymerase chain reaction (qRT-PCR). The sequences of the primers for *MAP1LC3B*, *BECN1*, and *SQSTM1* PCR amplification were 5′-CCGACTTATTCGAGAGCAGCATCC-3′ (forward) and 5′-GTCCGTTCACCAACAGGAAGAAGG-3′ (reverse) (*MAP1LC3B*, 24 bp), and 5′-GGAGCTGCCGTTATACTGTTCTGG-3′ (forward) and 5′-TGCCTCCTGTGTCTTCAATCTTGC-3′ (reverse) (*BECN1*, 24 bp), and 5′-CCGTCTACAGGTGAACTCCAGTCC-3′ (forward) and 5′-AGCCAGCCGCCTTCATCAGAG-3′ (reverse) (*SQSTM1*, 21 bp), respectively. All experiments were performed in triplicate. Human *GAPDH* cDNA was amplified as an internal control.

### Confocal immunofluorescence staining and analysis

The cells were cultured on cover slips in the 24-well plates for mRFP-GFP-LC3B adenovirus (Hanbio Co., Ltd., Shanghai, China) transfection. After being transiently transfected with mRFP-GFP-LC3 adenovirus for 6 h, cells were washed with PBS buffer and then treated as previously described. After 48-h treatment, cells were washed with PBS and fixed with 4% paraformaldehyde. The cells were stained with 4′,6-diamidino-2-phenylindole (DAPI) dye for 5 min and blocked with glycerol. Finally, the cells were analyzed by using a Zeiss LSM 880 confocal microscope (Carl Zeiss, Oberkochen, Germany).

### Statistical analyses

Each experiment was performed independently at least three times. The data are expressed as the mean ± standard deviation. Differences among different groups were analyzed by one-way analysis of variance (ANOVA), and significance was determined by using Bonferroni’s correction for multiple comparisons with independent sample *t* test. A two-sided *P* value of less than 0.05 was considered to be significant. The data were analyzed by SPSS 21.0 software (IBM Corporation, Armonk, NY, USA).

## Results

### Podocyte autophagic level differs among different classifications of lupus nephritis

**Fig. 1:**
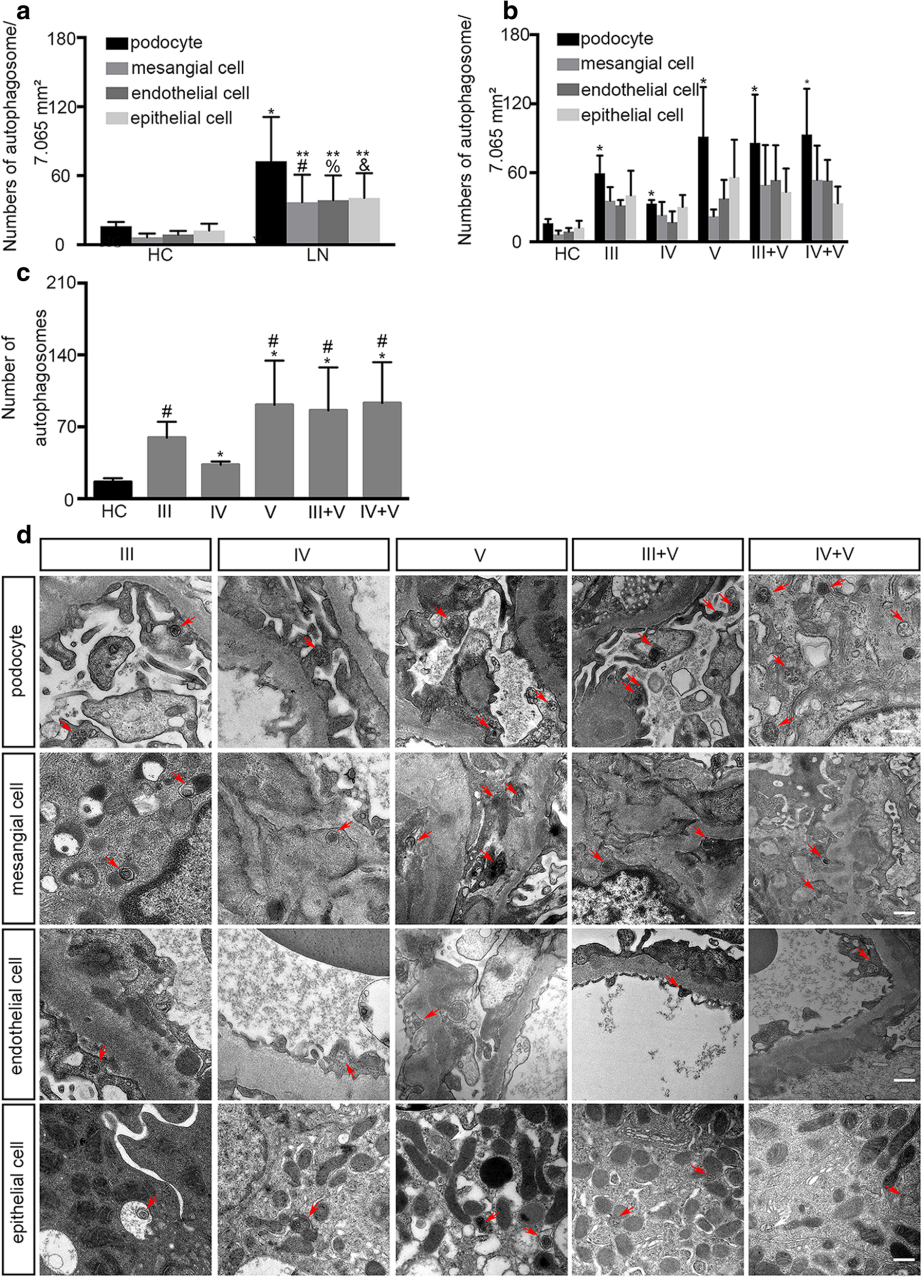
Numbers of autophagosomes of patients with lupus nephritis (LN) and healthy controls. Original magnification 25,000×. **a** Numbers of autophagosomes in podocytes, mesangial cells, endothelial cells, and epithelial cells of total patients with LN (n = 25) and healthy controls (n = 7); autophagosomes were counted under transmission electron microscope (TEM) in an area of copper mesh (approximately 7065 square millimeters). *P <0.05 versus control podocytes (n = 25); ^#^*P* <0.05 versus control mesangial cells (n = 25); ^%^*P* <0.05 versus control endothelial cells (n = 25); ^&^*P* <0.05 versus control epithelial cells (n = 25); **P <0.01 versus LN podocytes (n = 25). **b** Numbers of autophagosomes in podocytes, mesangial cells, endothelial cells, and epithelial cells of different classifications of patients with LN and healthy controls (n = 7). *P <0.05 versus control podocytes (n = 25). **c** Numbers of podocytes of patients with LN and healthy controls. *P <0.05 versus class III LN; ^#^*P* <0.05 versus class IV LN. **d** Representative images of autophagosome in renal resident cells in different classifications of LN are shown. The red frames label autophagosomes (scale bar 500 nm, original magnification 25,000×). All statistical analyses were performed by using SPSS version 21.0 (SPSS Inc., Chicago, IL, USA) and GraphPad Prism 6 (GraphPad Software, Inc., La Jolla, CA, USA) for macOS. A *P* value of less than 0.05 was considered statistically significant. Data were expressed as mean ± standard deviation (SD) and median with range or frequency, as appropriate. Intergroup comparisons were made by using Pearson chi-squared test for categorical variables and Student’s *t* test, Mann–Whitney *U* test, or Wilcoxon test for continuous variables, as appropriate. Abbreviation: *HC* healthy control

Compared with healthy volunteers (n = 7), the numbers of autophagosome in a glomeruli in an area of a copper mesh (approximately 7065 square millimeters) of podocyte, mesangial cell, and endothelial cell in patients with LN were different as well as in a proximal renal tubule in an area of a copper mesh of renal tubular epithelial cell, and the differences were significant (*P* <0.05) (Fig. 1a). Patients with LN had an increased autophagic activity level compared with that in the healthy volunteers, especially in podocytes.

Significant differences were observed among different types of LN regarding the numbers of autophagosome in podocytes (Fig. 1b, c). Among proliferative types of LN, V, III + V, and IV + V types demonstrated higher podocyte autophagic levels than other types (Fig. 1b, *P* <0.05).

### Podocyte autophagic level differs among patients with different 25(OH)D3 concentration

**Fig. 2:**
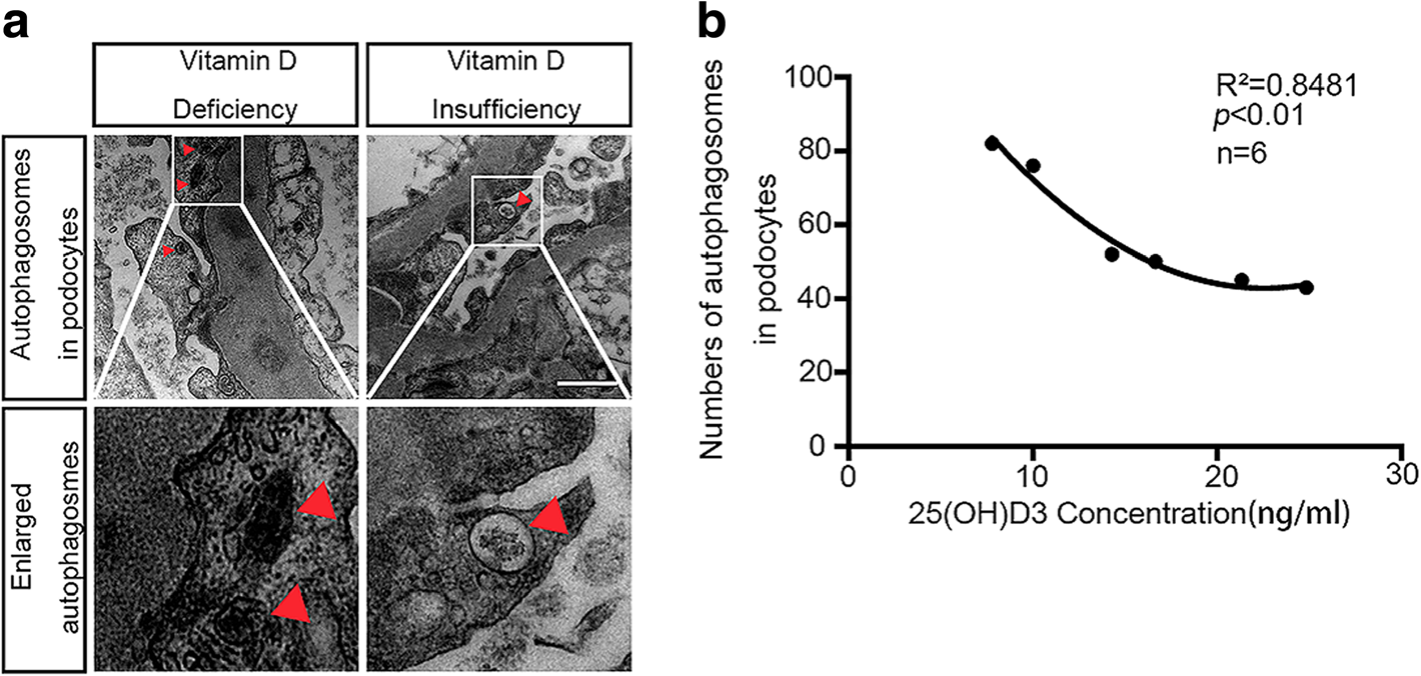
Autophagosomes in podocytes of lupus nephritis (LN) patients with vitamin D deficiency and vitamin D insufficiency (n = 6). **a** The red triangles label autophagosome, and enlarged autophagosomes are shown (scale bar 500 nm). Original magnification 25,000×. **b** The scatter plot and fitting curve are shown

Six patients with LN were observed with their serum 25(OH)2D3 concentrations and autophagosome numbers in podocytes under TEM. Serum 25(OH)D3 levels less than 30 and less than 20 ng/mL were defined as vitamin D insufficiency and vitamin D deficiency, respectively. Higher numbers of autophagosomes in patients with vitamin D deficiency were observed compared with patients with vitamin D insufficiency (Fig. 2 and Table 2).

**Table 2:** Six patients whose serum vitamin D concentrations were measured and autophagosomes numbers in podocytes were count under transmission electron microscope

Patient	Serum 1,25(OH)2D3 concentration, ng/mL	Numbers of autophagosome podocytes
1	7.79	82
2	10	76
3	14.29	52
4	16.66	50
5	21.34	45
6	24.84	43

### Vitamin D regulates autophagy level and protects podocytes from injury

Based on clinical findings, we investigated the role of autophagic activity in podocytes when incubated with IgG purified from serum of patients with LN through measuring the expression of autophagy-associated proteins, including LC3B, Beclin 1, p62, and podocyte marker protein, nephrin.

**Fig. 3:**
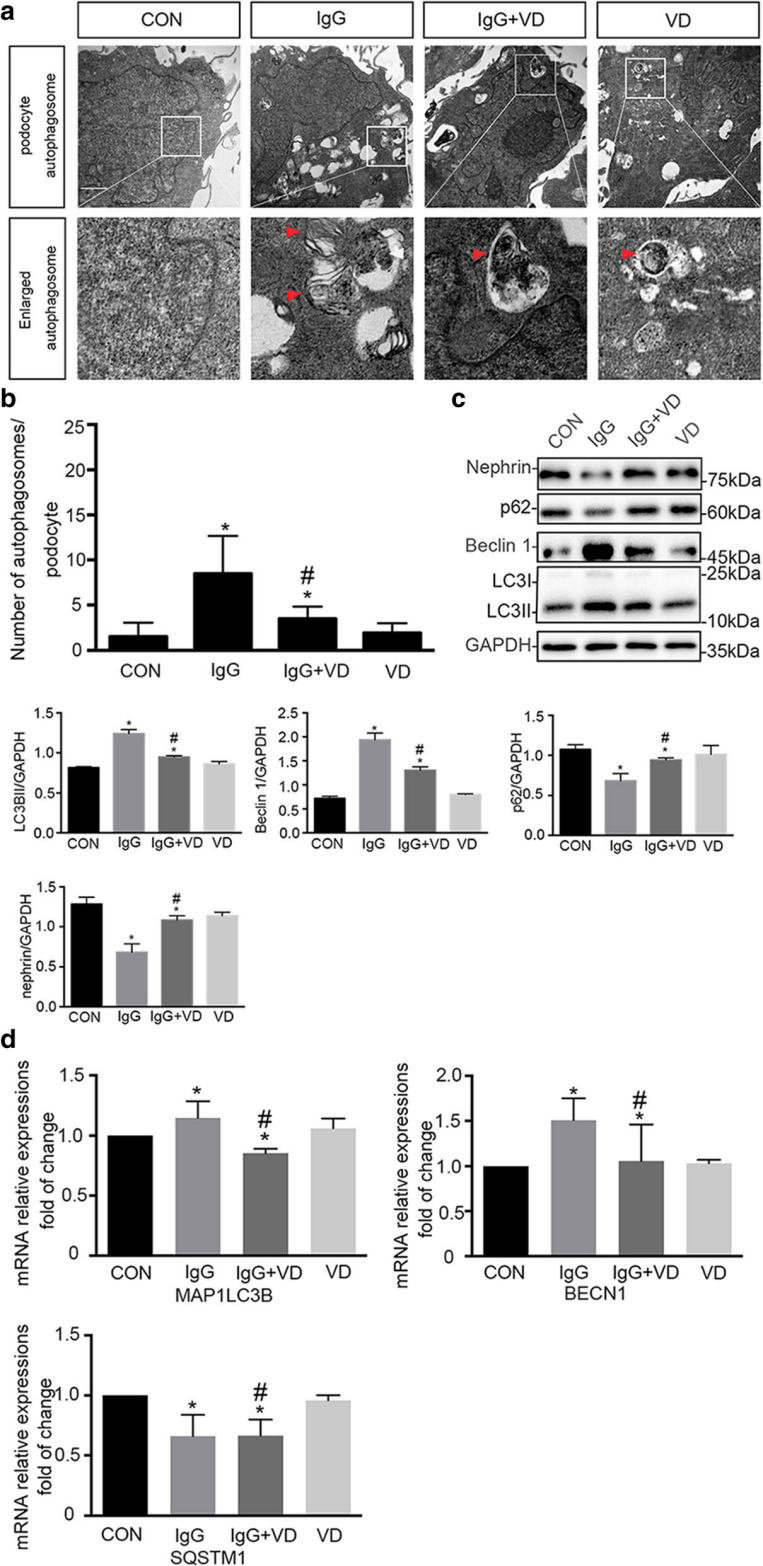
Autophagy in human podocytes (HPCs). **a**, **b** Induction of autophagy in HPCs by immunoglobulin G (IgG) purified from lupus nephritis (LN) patients’ serum or a combination of IgG and vitamin D or vitamin D alone. Observation of autophagosomes under transmission electron microscope (TEM). Numbers of autophagosome in 30 random HPCs under TEM were counted. All statistical analyses were performed by using SPSS version 21.0 (SPSS Inc., Chicago, IL, USA) and GraphPad Prism 6 (GraphPad Software, Inc., La Jolla, CA, USA) for macOS. A *P* value of less than 0.05 was considered statistically significant. Data were expressed as mean ± standard deviation and median with range or frequency, as appropriate. Intergroup comparisons were made by using Pearson chi-squared test for categorical variables and Student’s *t* test, Mann–Whitney *U* test, or Wilcoxon test for continuous variables, as appropriate. *P <0.05 versus control (CON) group; ^#^*P* <0.05 versus IgG group (n = 30). **c** Western blot analysis of LC3B, Beclin 1, p62, and nephrin expression in HPCs by IgG purified from serum from patients with LN or a combination of IgG and vitamin D or vitamin D alone. **d** Real-time polymerase chain reaction of *MAP1LC3B*, *BECN1*, and *SQSTM1* gene expressions was analyzed (n = 4). *P <0.05 versus CON group; ^#^*P* <0.05 versus IgG group. Abbreviation: *VD* vitamin D

IgG treatment elicited autophagy, shown by immunoblot analysis for upregulation of LC3B-II and Beclin 1 as well as the consumption of p62 (Fig. 3c). This was concomitant with podocyte injury, marked by reduced expression of nephrin. Vitamin D reduced aberrant autophagy after IgG treatment, resulting in protection against podocyte autophagy. This beneficial effect of vitamin D was associated with downregulated expression of autophagy-associated proteins. Real-time qRT-PCR revealed the changes in *MAP1LC3B*, *BECN1*, and *SQSTM1* genes and this was identical to the tendency in protein level (Fig. 3d).

Consistent with these observations, TEM imaging revealed a significant increase in the autophagic double-membrane compartments containing lamellar structures in the IgG group compared with the control group. During incubation with IgG (1.5 mg/mL) and vitamin D (100 nM), a significant decrease in the autophagic double-membrane compartments was observed (Fig. 3a). More importantly, we randomly selected 30 podocytes from each group for a statistic analysis. The results showed significant differences with regard to the numbers of autophagosomes among different groups (Fig. 3b, *P* <0.05).

**Fig. 4:**
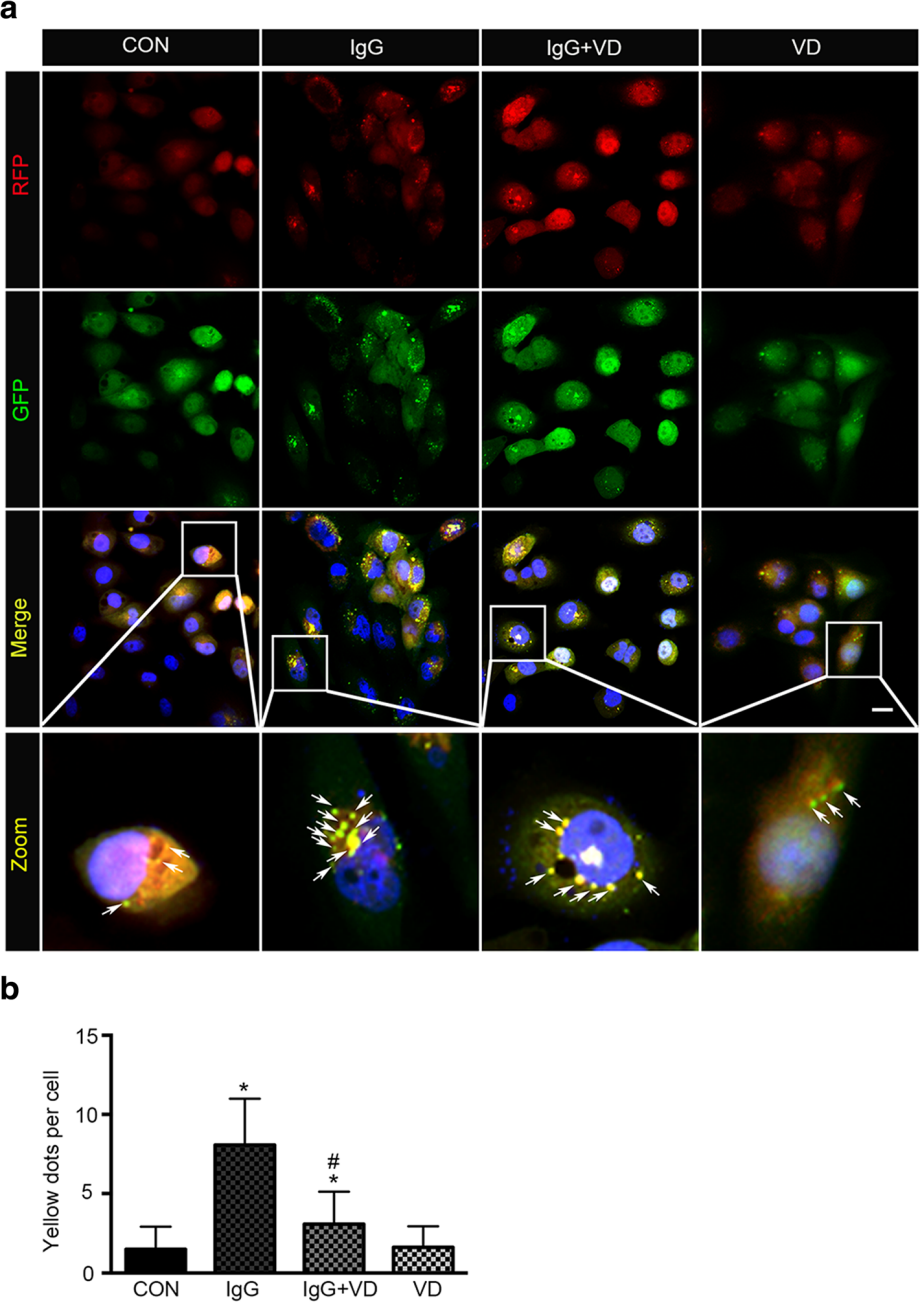
RFP-GFP-LC3 adenovirus which labeled LC3 was transfected into human podocytes (HPCs), and representative images of immunofluorescence were detected by laser scanning confocal microscope. Cells were counterstained with 4,6-diamidino-2-phenylindole (DAPI; blue). Autophagosomes (GFP+RFP+LC3 puncta) were labelled with yellow (merge from green and red). The zoom area enlarges representative images of human podocytes. Arrowheads indicate autophagosomes in human podocytes. Original magnification 400×. By detecting and analyzing the number of yellow dots, autophagic activity can be measured. Numbers of yellow dots in 30 random HPCs under transmission electron microscope were counted. All statistical analyses were performed by using SPSS version 21.0 (SPSS Inc., Chicago, IL, USA) and GraphPad Prism 6 (GraphPad Software, Inc., La Jolla, CA, USA) for macOS. A P value of less than 0.05 was considered statistically significant. Data were expressed as mean ± standard deviation and median with range or frequency, as appropriate. Intergroup comparisons were made by using Pearson chi-squared test for categorical variables and Student’s t test, Mann–Whitney U test, or Wilcoxon test for continuous variables, as appropriate. *P <0.05 versus control (CON) group; #*P*<0.05 versus immunoglobulin G (IgG) group (*n* = 30)

The accumulation of autophagosomes suggests an active ongoing autophagic process. Therefore, we next examined LN IgG-induced autophagy flux by using fluorescence confocal microscopy in human podocytes transfected with an adenovirus encoding the tandem fluorescent mRFP-GFP-LC3 (Fig. 4a). Under confocal microscopy, mRFP dots were red while GFP dots were green. In the merged images, autophagosomes and autolysosomes were labeled with yellow and red dots, respectively. Compared with the control group, the numbers of red and yellow dots were markedly increased after treatment with LN IgG. However, the number of autophagosomes and autolysosomes slightly increased after treatment with vitamin D alone compared with the control group. More importantly, we randomly selected 30 podocytes from each group for a statistic analysis. The results showed significant differences with regard to the numbers of yellow dots among different groups (Fig. 4b, *P* <0.05).

### Podocyte autophagic activity correlates with clinical data

**Fig. 5:**
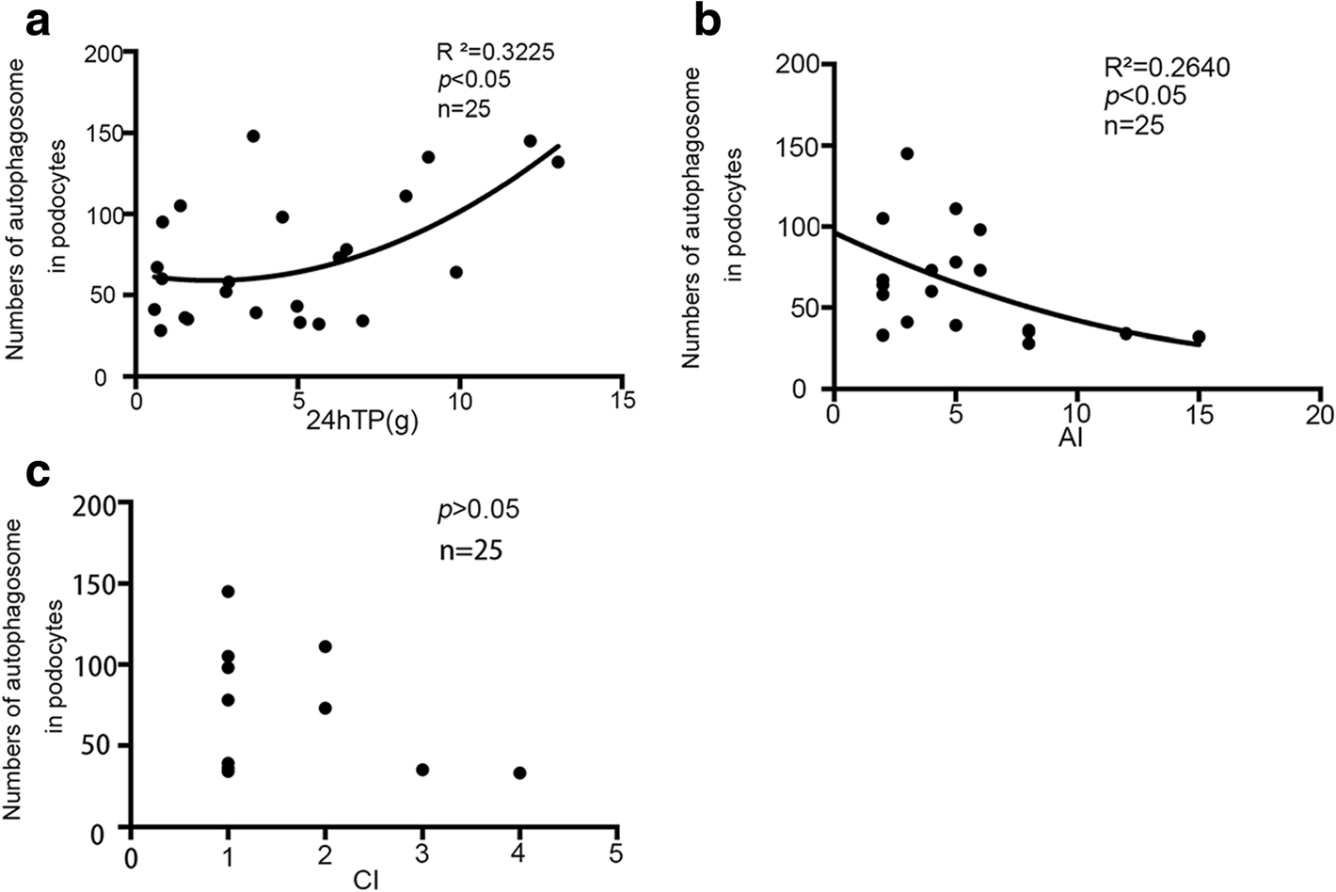
Podocyte autophagic activity correlates with clinical data. Numbers of autophagosomes in podocytes correlate with 24-h total protein in urine and active index. There is no correlation between numbers of autophagosomes in podocytes and chronic index. The scatter plot and fitting curve are shown

We then investigated the correlation between the number of autophagosome in podocytes and clinical findings. Pearson correlation was used in SPSS 21.0.0. In the clinical data, 24-h total protein was correlated with the number of autophagosome in podocytes (*P* <0.05), and the correlation coefficient was 0.505 (Fig. 5a). In the pathological data, AI was negatively correlated with the number of autophagosome in podocytes (*P* <0.05), and the correlation coefficient was −0.5072. Analysis of additional and relevant data did not demonstrate significant differences among different groups and these included SLEDAI, CRP, ESR, serum C3 and C4, CI, BUN, Scr, UA, serum albumin, GFR, hemoglobin, and urinary erythrocyte and urinary leukocyte.

## Discussion

Previous studies have demonstrated that autophagy plays a role in the pathogenesis of LN by regulating B cells, T cells, dendritic cells, phagocytes, and podocytes [14]. When stimulated by anti-dsDNA autoantibodies isolated from patients with LN, phagocytosis of immune complex occurs in podocytes, forming cytosolic speckles [20]. These aggregates harmed podocyte survivals and could be degraded by autophagy. He et al. [22] have demonstrated that podocyte autophagic activity is involved in the pathogenesis of LN and made an observation through different classifications of LN. However, the link is missing between clinical observations and mechanistic studies. In addition, autophagy functions in the protective role of vitamin D in podocytes in LN were not explored.

A previous study showed that IgG purified from the serum of patients with LN caused podocyte injury through inducing autophagy and that 1,25(OH)2D3 alleviated podocyte injury by reducing excessive autophagy. The injury of human podocytes caused by IgG purified from LN patients was alleviated by the downregulated LC3B and Beclin 1 and upregulated p62. LN IgG and vitamin D co-treatment resulted in fewer autophagosomes and autolysosomes compared with the LN IgG group, suggesting that vitamin D regulated autophagic flux. Collectively, these results suggest that vitamin D could effectively regulate autophagy response induced by IgG purified from patients with LN.

Renal involvement is common in SLE. Auto-antibodies deposited in the kidneys, leading to LN, which eventually can progress to end-stage renal failure. Winfield et al. [23] demonstrated that the affinity of circulating anti-dsDNA antibodies to dsDNA correlated with the activity of nephritis. Additionally, the anti-dsDNA activity in IgG fractions eluted from nephritic glomeruli was higher than that in corresponding serum samples [23]. Podocytes, or glomerular visceral epithelial cells, have minimal capacity for proliferation and regeneration under steady-state conditions. They are highly specialized cells involved in the charge and size filtration characteristics of the glomerulus. When stimulated with pathogens, podocytes might protect themselves through autophagy [24]. In this study, cultured human podocytes were treated primarily by autoantibodies from serum from patients with LN, inducing autophagy flux and producing foot process injury. Proteins expressed by podocytes are important for the integrity of the filtration barrier. Among them, nephrin locates specifically in the slit diaphragm region of the podocyte foot processes and can be used as a marker of podocytes [25]. It plays a role in the structural integrity of the slit and its absence results in massive proteinuria [25].

As an intracellular protective mechanism, autophagy has received widespread attention. Vitamin D may regulate the autophagy process, possibly through modulating effects on both the innate and adaptive immunity [26]. Previous studies have demonstrated the association between vitamin D and autophagy in autoimmune diseases [27] and shown that vitamin D may prevent podocytes from injury in nephritis and nephropathy [9, 28]. Our results indicate that vitamin D is involved in the autoantibody interference with the process of podocyte repair through the autophagy pathway. Under pathological conditions, upregulation of vitamin D is associated with downregulation of autophagy, thereby protecting injured podocytes. Under normal conditions, upregulation of vitamin D may be associated with upregulation of autophagy, thus facilitating podocyte injury.

It was apparent that the numbers of autophagosomes in podocytes, mesangial cells, endothelial cells of glomeruli, and renal tubular epithelial cells in the proximal renal tubule of patients with LN were higher than those in healthy volunteers, particularly in podocytes than in other cells, consistent with previous reports that injured podocytes display a relatively high level of autophagic activity except class II LN [24]. We found that the number of autophagosomes in podocytes from class V LN was higher than that in healthy volunteers, somewhat in contradiction to the observations made by He et al. [24]. This discrepancy may be explained by different methods to count the numbers of the autophagosomes. When counting autophagosome numbers in podocytes under TEM, we obtained the sum by counting all the podocytes, including primary and secondary foot processes in the same area of a copper mesh. In contrast, He et al. randomly counted 30 podocytes under TEM. Furthermore, it is difficult to identify the exact podocytes and their own foot processes. On the other hand, in order to relieve environmental stress, podocytes from control patients (with renal carcinoma) may experience a relatively higher basal level of autophagic activity than the healthy. Since control patients were different from different area, autophagic level may differ.

In different classifications of LN, we investigated proliferative LN, pure membranous LN, and the combination of proliferative and membranous LN and excluded class I and II LN. In class I and II LN, mesangial immune deposits with or without mesangial proliferation that is evident under light microscopy but not evident under TEM. The lesions are not that serious and often are not accompanied by acute nephritic syndrome or heavy proteinuria. In proliferative LN, pure membranous LN, and the combination of proliferative and membranous LN, autoantibodies deposits more or less in GBM (glomerular basement membrane), as described previously, podocytes take part in the formation of GBM, which is involved in the pathology of LN.

## Conclusion

Our results suggest that podocyte autophagy plays a role in the pathogenesis of LN and is associated with proteinuria as well as disease activity. Moreover, vitamin D has a protective role in podocyte injury in patients with LN by regulation of autophagic activity. In our follow-up studies, we will further dissect the detailed mechanism underlying podocyte autophagy. In conclusion, our results provided new insights into the function of vitamin D in podocyte injury in the pathogenesis of LN.

## Abbreviations

AI: Active index
BUN: Blood urea nitrogen
cDNA: Complementary DNA
CI: Chronic index
CRP: C-reactive protein
ESR: Erythrocyte sedimentation rate
GBM: Glomerular basement membrane
GFR: Glomerular filtration rate
HPC: Human podocyte
IgG: Immunoglobulin G
LN: Lupus nephritis
PBS: Phosphate-buffered saline
qRT-PCR: Quantitative reverse transcription-polymerase chain reaction
Scr: Serum creatinine
SLE: Systemic lupus erythematosus
TEM: Transmission electron microscope
UA: Urea acid
